# Supplementary figures and images for: Functional genome-wide siRNA screen identifies KIAA0586 as mutated in Joubert syndrome
Source: eLife. 2015 May 30;4:e06602. doi: 10.7554/eLife.06602 (PMC4477441; doi:10.7554/eLife.06602)

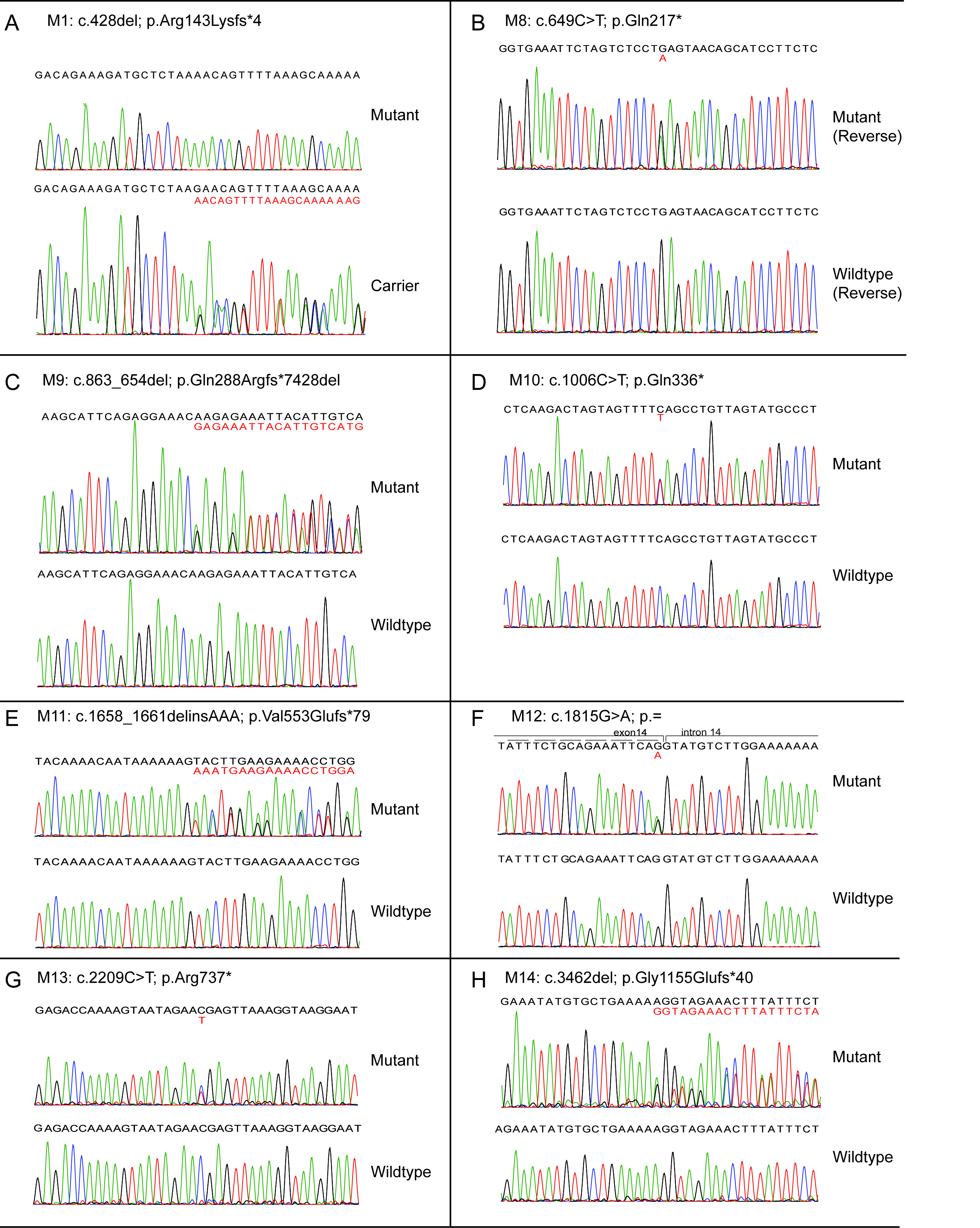

Supplement: Table 1—source data 1. — Chromatograms of mutations in the KIAA0586 gene identified in the additional cohort of Mediterranean individuals with Joubert syndrome. DOI: http://dx.doi.org/10.7554/eLife.06602.012 [file elife06602s001.tif]
