# Supplementary material for: Functional genome-wide siRNA screen identifies KIAA0586 as mutated in Joubert syndrome
Source: eLife. 2015 May 30;4:e06602. doi: 10.7554/eLife.06602 (PMC4477441; doi:10.7554/eLife.06602)
Supplement: Supplementary file 1. — Parameter output genome-wide siRNA analysis screen. Table listing all parameters used in the analysis of the genome wide siRNA screen. DOI: http://dx.doi.org/10.7554/eLife.06602.015 [file elife06602s002.docx]

**Supplementary file 1. Parameter output genome wide siRNA analysis screen**

| **Parameter output** | **Definition parameter** |
| --- | --- |
| Number of cells analyzed | The total cells in the well |
| Area of the nucleus | Average area of nuclei for the well |
| Total nuclei Intensity | Total integrated intensity of the nucleus averaged over all fields/cells in the well |
| Average nuclei Intensity | Average intensity of the nucleus averaged over all fields/cells in the well |
| Roundness nuclei | Average roundness of the nuclei for all cells in the well |
| Perimeter nuclei | Average perimeter of the nuclei for all cells in the well |
| Elongation nuclei | Average elongation of the nuclei for all cells in the well |
| Red total nuclear Intensity | Total integrated intensity of the nucleus in the red channel |
| Red average nuclear Intensity | Average nuclear intensity in the red channel |
| Green total cytoplasmic Intensity | Integrated cytoplasm intensity of the cell averaged for all fields/cells in the well |
| Green average cytoplasmic Intensity | Average cytoplasm intensity of the cell averaged for all fields/cells in the well |
| Green total cell intensity | Integrated cell intensity averaged for all cells in the well |
| Green Average cell intensity | Average cell intensity averaged for all cells in the well |
| Number of cilia per well | Total number of cilia in the whole well |
| Total cilia Intensity | Total integrated intensity of cilia |
| Average cilia Intensity | Average intensity of the cilia |
| Number of cilia per cell | Average number of cilia per cell |
| Area per cilia | Average area of the cilia |
| Number cells with cilia | Number of cells having at least one cilia |
| Number of cells with more than 1 cilia | Number of cilia having more than one cilia |
| Number of cells with more than 2 cilia | Number of cells having more than two cilia |
| Number or red cells | Total red cells in the well |
| Percent hit for red cells | Number of cells having higher intensity in the red channel (Red Cells) |
| Number of cilia per red cell | Average number of cilia per red cell |
| Number of red cells with cilia | Number of red cells having at least one cilia |
| Number of red cells with more than 1 cilia | Number of red cells having more than one cilia |
| Number of red cells with more than 2 cilia | Number of red cells having more than two cilia |
| Ratio of cilia for red cells to cilia for all cells | Average ratio for number of cilia for red cells to the number of cilia for all the cells in the well |
| Percent of total Red Cells with Cilia | Number of red cells having one cilia |
| Percent of ciliated cells | Number of cells having one cilia |
| Percent of Red cells | Number of red cells from total cells |
