# Supplementary material for: Functional genome-wide siRNA screen identifies KIAA0586 as mutated in Joubert syndrome
Source: eLife. 2015 May 30;4:e06602. doi: 10.7554/eLife.06602 (PMC4477441; doi:10.7554/eLife.06602)
Supplement: Supplementary file 4. — Clinical features and KIAA0586 mutations. (A) Clinical features of individuals with KIAA0586 mutations. (B) Nomenclature per isoform of the identified KIAA0586 mutations. DOI: http://dx.doi.org/10.7554/eLife.06602.018 [file elife06602s005.docx]

**Supplementary file 4A. Clinical features of individuals with *KIAA0586* mutations**

| **Family ID** | **MTI-103** | | | **MTI-165** | **MTI-233** | **MTI-505** | **MTI-1944** | **COR354** | | | |
| --- | --- | --- | --- | --- | --- | --- | --- | --- | --- | --- | --- |
| Individual ID | 3-1 | 3-2 | 3-3 | 2-1 | 2-1 | 2-1 | 2-1 | 2-1 | | 2-2 | 2-3 |
| Country of origin | USA | USA | USA | Mexico | USA | Syria | Turkey | Turkey | | Turkey | Turkey |
| Gender | F | F | F | M | M | F | M | M | | M | M |
| Consanguinity | - | - | - | - | - | + | - | + | | + | + |
| **Neurological Findings** |  |  |  |  |  |  |  |  | |  |  |
| Hypotonia | + | + | + | + | + | + | + | + | | + | + |
| Ataxia | + | + | - | + | + | + | NA | + | | + | NA |
| Psychomotor delay | + | + | + | + | + | + | + | + | | + | + |
| Intellectual disability | + | + | + | NA | + | + | + | + | | + | + |
| Breathing abnormalities | - | - | Apnea as newborn | Apnea | + | - | NA | - | | - | - |
| Seizures | - | + | - | - | + | - | NA | - | | - | - |
| Macrocephaly | - | - | + | NA | - | + | NA | - | | - | - |
| **Ophthalmological Findings** |  |  |  |  |  |  |  |  | |  |  |
| Retinopathy | - | - | - | - | - | - | NA | - | | - | - |
| Coloboma | - | - | - | Unilateral | - | - | NA | - | | - | - |
| Oculomotor apraxia | - | - | - | + | + | - | NA | + | | + | NA |
| Nystagmus | - | - | - | - | + | - | NA | + | | - | NA |
| Other | Duane's  syndrome | - | - | - | - | - | NA | peripapillary pigmentary ring | | excavatio papillae |  |
| **Miscellaneous Findings** |  |  |  |  |  |  |  |  | |  |  |
| Nephronophthisis | NA | NA | NA | - | - | - | NA | - | | - | - |
| Hepatic fibrosis | NA | NA | NA | - | - | NA | NA | - | | - | - |
| Polydactyly | - | - | - | - | - | - | NA | - | | - | - |
| Obesity | - | - | - | - | - | - | NA | - | | - | - |
| Other | - | - | - | - | - | - | NA | - | | - | - |
|  |  | | |  |  |  |  |  | | | |
|  |  | | |  |  |  |  |  | | | |
|  |  | | |  |  |  |  |  | | | |
|  |  | | |  |  |  |  |  | | | |
|  |  | | |  |  |  |  |  | | | |
| **Family ID** | **MTI-103** | | | **MTI-165** | **MTI-233** | **MTI-505** | **MTI-1944** | **COR354** | | | |
| Individual ID | 3-1 | 3-2 | 3-3 | 2-1 | 2-1 | 2-1 | 2-1 | 2-1 | 2-2 | | 2-3 |
| **Cranial MRI Findings** |  |  |  |  |  |  |  |  |  | |  |
| Molar Tooth sign | + | + | + | N/A | + | + | + | + | + | | + |
| Cerebellar vermis dysgenesis | + | + | + | N/A | + | + | + | + | + | | + |
| Hypoplasia of the brainstem | - | - | - | N/A | + | + | - | + | + | | + |
| Occipital meningocele | - | - | - | N/A | - | - | - | - | - | | - |
| Thin corpus callosum | + | - | - | N/A | - | + | - | + | + | | - |

The following abbreviation is used: F = Female, M = Male, NA = not available

**Supplementary file 4B. Nomenclature per isoform of the *KIAA0586* mutations by whole exome sequencing**

|  | **M1** | **M2** | **M3** | **M4** | **M5** | **M6** | **M7** |
| --- | --- | --- | --- | --- | --- | --- | --- |
| **T1: NM_001244189.1** | c.428del | c.1120+1G>A | c.1413-1G>C | c.293T>C | c.1413-?_2793+?del | c.2414-1G>C | c.74del |
|  | p.Arg143Lysfs*4 | p.Thr323Hisfs*3 | p.Arg471Serfs*2 | p.Met98Thr | p.? | p.? | p.Lys25Argfs*6 |
| **T2: NM_001244190.1** | c.347del | c.916+1G>A | c.1209-1G>C | c.212T>C | c.1209-?_2589+?del | c.2210-1G>C | c.-8del |
|  | p.Arg116Lysfs*4 | p.? | p.Phe404Lysfs*50 | p.Met71Thr | p.? | p.? | p.? |
| **T3: NM_14749.3** | c.392del | c.541+1G>A | c.1254-1G>C | c.257T>C | c.1254-?_2406+?del | c.2027-1G>C | c.38del |
|  | p.Arg131Lysfs*4 | p.? | p.Arg418Serfs*2 | p.Met86Thr | p.? | p.? | p.Lys13Argfs*6 |
| **T4: NM_001244191.1** | c.137del | c.706+1G>A | c.999-1G>C | c.2T>C | c.999-?_2379+?del | c.2000-1G>C | - |
|  | p.Arg46Lysfs*4 | p.? | p.Arg333Serfs*2 | p.Met1? | p.? | p.? | - |
| **T5: NM_001244192.1** | c.137del | c.829+1G>A | c.1122-1G>C | c.2T>C | c.1122-?_2502+?del | c.2123-1G>C | - |
|  | p.Arg46Lysfs*4 | p.? | p.Arg374Serfs*2 | p.Met1? | p.? | p.? | - |
| **T6: NM_001244193.1** | - | c.541+1G>A | c.834-1G>C | - | c.834-?_2214+?del | c.1835-1G>C | - |
|  | - | p.? | p.Arg278Serfs*2 | - | p.? | p.? | - |
