# Supplementary material for: Functional genome-wide siRNA screen identifies KIAA0586 as mutated in Joubert syndrome
Source: eLife. 2015 May 30;4:e06602. doi: 10.7554/eLife.06602 (PMC4477441; doi:10.7554/eLife.06602)
Supplement: Supplementary file 5. — Primers. (A) Primers for KIAA0586 mutation confirmation and segregation analysis. (B) Primers for KIAA0586 mRNA and expression analysis. DOI: http://dx.doi.org/10.7554/eLife.06602.019 [file elife06602s006.docx]

**Supplementary file 5A. Primers for *KIAA0586* mutation confirmation and segregation analysis**

| ***KIAA0586*** | **Forward** | **Reverse** | **Product size (bp)** |
| --- | --- | --- | --- |
| M1-specific | TTCGATCGTGGAATGCTATG | TGTCTAGCACCATGAGAAATGC | 341 |
| M2-specific | CCTCCAGAGCAGTGGAAAAG | CAAGGTAGCCTGAGAAACACAA | 287 |
| M3-specific | TTTTCCTGCTCCTTTTCTGC | TTCTCTTTTTGGCCAAGGTC | 338 |
| M4-specific | TGCATGCAAATTGTCTTTCC | CAAGGCATGCTGCTAATCAA | 294 |
| M6-specific | GGGGTGATTGTCAGATAGGG | CCTGCACAGTAAGCTGAGGA | 313 |
| M7-specific | TCAGCTTTGTGGATGTTCGAC | GCTTCCTGGCCATTTTCCC | 477 |
| Exon 1 | TGTCCGGAGTTTGTTTCCAC | GTCAACACCCACTTCCCTTC | 143 |
| Exon 2 | AGACTGAGTGGGATTAATGGG | TAGGCTTCCTGGCCATTTTC | 557 |
| Exon 3 | TTGTCTTTCCAACTTCTGCG | AAACAAGGCATGCTGCTAATC | 287 |
| Exon 4 | AATACACAGTCCTGCCCTCC | ACAAAGCTCTGGATATGAAAGG | 328 |
| Exon 5 | TTCGATCGTGGAATGCTATG | GTGTCTAGCACCATGAGAAATG | 342 |
| Exon 6 | GTGAAGTGATGGGTATGCTAATAGC | CAGGTGCAGTGGCTAGCACATGT | 477 |
| Exon 7 | CACCTTCGGCAGATTACCAT | CAAATTGAGAGCAACTACAATGTTC | 389 |
| Exon 8 | TCTGCCTTTCATTTCTGTGAC | GAGTTACCCGCATTACAGGAG | 559 |
| Exon 9 | CTTTCCAATGGCATCACTGC | AGCACAAAGAAAATTCACAGC | 388 |
| Exon 10 | CACCGCAGCTTCAGTAGTTC | AAGAAGCTTGATTTTCCCAGC | 551 |
| Exon 11 | AGGCCAGAATACAAATATGTGAG | TTTTGGCTAGCCTCATTTTATC | 540 |
| Exon 12 | GCATAACCTTTTCCTGCTCC | ATGGCTCAGTCCTGGTCAAG | 577 |
| Exon 13 | ATGCCTGGCCCACATTAG | GGGGCCTAATATCCTTCATC | 770 |
| Exon 14 | AGATCTGATCCAAGTCCTGCTC | TGCTCCTAAATCCACTGTCTTC | 446 |
| Exon 15 | CATTCCCCACTCCAAAAGATAC | AAACACCTTCACATTCGCTTC | 547 |
| Exon 16 | AAGGGCTGTTGAAAACAGAG | TTTTCCATCCCCAAGCATC | 447 |
| Exon 17 | GTTTTGGGGCCTTGTAACC | GTTCCAGACCACTCTGAGCC | 432 |
| Exon 18 | GGGTGATTGTCAGATAGGGC | GAAGCAATATAATGGATTTCCG | 397 |
| Exon 19 | AAAACTTAAAGGGCATGCTTTG | GCAGGCACCTGTAATCCC | 523 |
| Exon 20 | TGGGGACTAGCTTGGATTTC | CCAAAGATAGTCAACAACCTGG | 326 |
| Exon 21 | GCTGTTGGTTAATTTAGAAGGG | CCTCCAACAATAGTTAAGCCC | 478 |
| Exon 22 | CAACATGTTTGTGGCATTGG | GCTGCAATATGAAATAAGAGGG | 418 |
| Exon 23 | AGCCTGGGTGACAGAGTGAC | AATGATCTTATGGTATGCAGCTT | 393 |
| Exon 24 | CTGCAGGCCAATCGTAGAC | CGCAATCAATTCCTAAATTTCC | 457 |
| Exon 25 | AAGCAATGTATCATGCCATCC | CATAGGCCATCTTGAAAGCTC | 646 |
| Exon 26 | CAAAATGAATACAGGCCTTCAG | AACTAAATTGCATTGAGTCCCC | 459 |
| Exon 27 | GATTGAGTAGTTTGTGAATTACTTG | AAACCTGATCAGTGGCAACC | 308 |
| Exon 28 | TTTGAGGCAAAGATAGAGGG | AAGGTGGTTGGAACAGATGG | 525 |
| Exon 29 | CCTTCCAAGGAGTCAAAGGG | AAAGAAATTTTGCAAGCCCC | 385 |
| Exon 30 | GCAGGTGAGAAATATTGGTGG | ACCACGCTAAGGCAACAGTG | 322 |
| Exon 31 | AAGAGTTTGCTGCCAAATTAAG | GGGTAAAGGAAAGACACCAGC | 714 |
| Exon 32 | GGGAGTGCATTTGGTTTGTG | GCTGAGTTTGTAACCTGGGG | 327 |
| Exon 33 | TCCGTCTCCCGTCTCTCTATT | TCTACTTGAGACTGGAGGGTGA | 234 |
| Exon 34 | AGCACCTTGGGACAAGAGAG | GCCATGCCCTTTCACTACTC | 555 |

**Supplementary file 5B. Primers for *KIAA0586* mRNA and expression analysis**

| **mRNA analysis** | **Forward** | **Reverse** |
| --- | --- | --- |
| Exon 8-10 (M2 analysis) | GAGTGATTTGGAAGCAAAAGTCAATTC | CTCTGTGACACGACACTTCCATATTTTC |
| Exon 10-12/13 (M3 analysis) | GAAAATATGGAAGTGTCGTGTCA | TCAGAGATTCTTTTGGCTGGAC |
| Exon 2/3-7 (T1) | GGAACATCACGTGGTTCATCA | CATCCTTCTCTATGCCTGCA |
| Exon 3/4-7 (T2, T3, T4) | ATGGTGTCAGAAAGTGATT | CATCCTTCTCTATGCCTGCA |
| Exon 13-16 (T1, T2, T4, T5) | TCGTGCAAAAGATGGAGCTG | TGCTTCGATGGCCCTGATAA |
| Exon 13-16 (T1, T2, T3, T4, T5) | GAAAATATGGAAGTGTCGTGTCA | TCAGAGATTCTTTTGGCTGGAC |
| *GAPDH* | GTGTTCCTACCCCCAATGTGT | ATTGTCATACCAGGAAATGAGCTT |
| Exon 9 fw | TGCTGCACTCAAGACTAGTAGT | CCAAGATTAGGGTGTTCTGGT |
| Exon 13 fw | GAAGCTTCCAGATCTTCCACA | CAGCTCCATCTTTTGCACGA |
| Exon 16 fw | TGTACACACAGGGGCTTTTG | TTGGTCTCTGTGGTCTGGAC |
| Exon 19 fw | ACAGCATTTCAAATAGTAGTG | TCCAAGTTTGCACAGGAGGA |
| Exon 23 fw | TGAGACCATTGCTGTCATGC | TCCCCATACCGGCAAATATGT |
| *GADPH* (for gDNA) | ATCATCCCTGCCTCTACTGG | GTCCACCACTGACACGTTG |

Primers with a slash (/) indicate primers at the exon-exon boundary from cDNA.
